# Evaluation of the natural killer cell subsets and their relationship with serum interferon gamma and vitamin D levels in women with stages III and IV endometriosis: A case-control study

**DOI:** 10.18502/ijrm.v22i7.16933

**Published:** 2024-09-12

**Authors:** Samira Najafi Chamgordani, Nafiseh Esmaeil, Maryam Hashemi, Afshin Amari, Maryam Seyedtabib, Mehri Ghafourian

**Affiliations:** ^1^Department of Immunology, School of Medicine, Ahvaz Jundishapur University of Medical Sciences, Ahvaz, Iran.; ^2^Department of Immunology, School of Medicine, Isfahan University of Medical Sciences, Isfahan, Iran.; ^3^Department of Obstetrics and Gynecology, School of Medicine, Isfahan University of Medical Sciences, Isfahan, Iran.; ^4^Cellular and Molecular Research Center, Medical Basic Sciences Research Institute, Ahvaz Jundishapur University of Medical Sciences, Ahvaz, Iran.; ^5^Department of Biostatistics and Epidemiology, School of Health, Ahvaz Jundishapur University of Medical Sciences, Ahvaz, Iran.; ^6^Fertility, Infertility and Perinatology Research Center, Ahvaz Jundishapur University of Medical Sciences, Ahvaz, Iran.

**Keywords:** Endometriosis, NK cells, IFN-gamma, Vitamin D.

## Abstract

**Background:**

Natural killer (NK) cells play a critical role in the pathogenesis of endometriosis. Moreover, a normal vitamin D level is remarkably associated with an optimal immune response. So, there may be a probable relationship between these factors and the endometriotic women.

**Objective:**

This study aimed to evaluate the percentage of NK cells and their subsets and their relationship with serum levels of vitamin D and interferon-gamma (IFN-γ) in women with endometriosis.

**Materials and Methods:**

In this case-control study, 29 women with stage III-IV endometriosis and 30 healthy controls were enrolled. The study was conducted in the Immunology Department of Isfahan University of Medical Sciences, Isfahan, Iran between November 2021 and June 2022. The percentage of NK cells and their subsets, including CD56 ${\;}^{\text{dim}}$ CD16 ${\;}^{+}$ , CD56 ${\;}^{\text{bright}}$ CD16 ${\;}^{-}$ and CD56 ${\;}^{\text{bright}}$ CD16 ${\;}^{\text{bright}}$ were measured in the peripheral blood samples using flow cytometry. Serum levels of vitamin D and IFN-γ were also measured using the enzyme-linked immunosorbent assay.

**Results:**

The mean percentage of NK cells in women with endometriosis increased significantly compared to the control group (p = 0.03). The percentage of CD56 ${\;}^{\text{dim}}$ CD16 ${\;}^{+}$ (p = 0.007) and CD56 ${\;}^{\text{bright}}$ CD16 ${\;}^{\text{bright}}$ (p = 0.043) increased significantly in women with endometriosis in comparison with the control group, but the percentage of CD56 ${\;}^{\text{bright}}$ CD16 ${\;}^{-}$ subset was not significantly different. No relationship was observed between NK cells and their subsets with vitamin D and IFN-γ in the studied groups.

**Conclusion:**

The study of NK cell subsets and their related factors can be useful in assessing and treating women suffering from endometriosis. However, more comprehensive studies are required to draw definitive conclusions about these observations.

## 1. Introduction

Almost 10% women of reproductive age have endometriosis, one of the most prevalent gynecological and estrogen-dependent disorders. This condition, which is characterized by tissue implants resembling endometrial glands and stroma on the visceral and peritoneal surfaces of the pelvis as well as on the ovaries and outside the uterine cavity (1), is linked to symptoms like dyspareunia, dysmenorrhea, dysuria, pelvic pain, and infertility (2). Physical examination, pelvic ultrasound, transvaginal ultrasound, and pelvic magnetic resonance imaging are some of the diagnostic procedures for endometriosis. However, laparoscopy is the only reliable and conclusive method for diagnosing endometriosis.

Hormone therapy, painkillers, and surgery are categorized as the most available treatments used for endometriosis (3). According to the reported studies, a dysregulated immune response is closely related to the occurrence of endometriosis. The phenotype and function of natural killer (NK) cells in the peritoneum, blood, and endometrium can influence the pathophysiology of endometriosis (4). Based on CD56 and CD16 expression, NK cells are divided into 2 groups: CD56 ${\;}^{\text{bright}}$ CD16 ${\;}^{-}$ NK cells and CD56 ${\;}^{\text{dim}}$ CD16 ${\;}^{+}$ NK cells. The cytokines, including interleukin (IL) 10, IL-13, tumor necrosis factor-alpha (TNF-α), and interferon-gamma (IFN-γ), are mostly produced by CD56 ${\;}^{\text{bright}}$ cells, which comprise 10% of all NK cells in the peripheral blood (PB) and are primarily found in the decidua and uterine endometrium (5).

On the other hand, 90% or so of all circulating PB NK cells are CD56 ${\;}^{\text{dim}}$ cells with strong cytotoxicity (2). In the past few years, phosphorus and calcium homeostasis regulation and the role of low vitamin D levels in the occurrence of various diseases of the female reproduction system, such as endometriosis, have been assessed (6).

It has been demonstrated that vitamin D is crucial for controlling immunological responses. Due to the immunoregulatory properties of vitamin D, this prohormone is also considered in endometriosis, as its function leads to the suppression of IL-1β and TNF-α expression in this condition (7). Regarding immune system dysfunction in women with endometriosis, cytokine imbalances have been reported. In this regard, the role of IFN-γ has been particularly highlighted in the severity and immunopathogenesis of endometriosis (6). Moreover, the vitamin D receptor is found on the surface of most immune cells, including lymphocytes, and it controls the production of cytokines, including IL-4, IL-5, IL-6, IL-10, IL-13, and IFN-γ (8). It may show that vitamin D is essential for the growth, maturation, differentiation, and cytotoxicity of NK cells, as well as their function (9).

Not much research has been done on how vitamin D affects immune cells in endometriosis-affected women's PB. The current study was designed to compare endometriosis cases to healthy individuals in terms of the percentage of NK cells and their subsets in PB and their relationship to the serum levels of vitamin D and IFN-γ.

## 2. Materials and Methods

### Study participants

In this case-control study, 59 women of reproductive age (18–45 yr) were enrolled. The study was conducted in the Immunology Department of Isfahan University of Medical Sciences, Isfahan, Iran between November 2021 and June 2022.

2 groups were included in the study: 29 women with late endometriosis (stages III and IV) according to the revised American Society for Reproductive Medicine classification (10) as the case group and 30 healthy women with at least one live birth, without clinical or para-clinical evidence of endometriosis, as the control group. A gynecologist chose the case group, among the individuals who referred to the Shahid Beheshti hospital in Isfahan, Iran. The inclusion criteria were those who complained of pelvic pain, including dysmenorrhea, dyspareunia, or dyschezia, and whose endometriosis was diagnosed by transvaginal or transrectal sonography. On an average, 20 severe cases of endometriosis come to this center every month.

The following were the exclusion criteria for both groups: The existence of known autoimmune disorders and other acute or chronic inflammatory disorders, diagnosis of malignant neoplasia and infectious disease, diagnosed systemic disease including liver, kidney, coronary disease, diabetes, and hypertension, hormonal treatment (including oral contraceptives, gonadotropin-releasing hormone analogs, etc.); vitamin D supplementation during the previous 3 months, pregnancy, and menopause. In both the follicular and luteal phases of the menstrual cycle, 5 ml of PB were drawn from each participant and divided into 2 parts: 2 ml of the blood was transferred to an ethylenediamine tetra acetic acid tube for flow cytometry. The remaining 3 ml was collected in a different tube and left to clot at room temperature. The serum was then separated and kept at -70 C until serum vitamin D and IFN-γ were measured.

### Sample size 

Based on a study with similar parameters (the disparity in the KIR2DL1 ${\;}^{\_}$ NK cells index between the case and control groups) (11), and considering a 95% confidence level and 80% test power, the sample size was estimated at 25 patients per group. Ultimately, we enrolled 30 patients in each group using the following formula. 


$$n=\frac{{\left(z_{1-\frac{\alpha }{2}}+z_{1-\beta }\right)}^{2}\left(\sigma_{1}^{2}+\sigma_{2}^{2}\right)}{{\left(\mu_{1}-\mu_{2}\right)}^{2}}$$

### Isolation of peripheral mononuclear cells (PBMC) and flow cytometry analysis

PBMCs were isolated by Ficoll density gradient centrifugation (density, 1.077 ± 0.002) (Sigma, USA) and washed 2 times with phosphate-buffered saline. PBMCs were then stained with FITC-Anti-CD3 (BioLegend, 344804), PerCP-Anti-CD16 (BioLegend, 360719), and PE-Anti-CD56 (BioLegend, 362508), and incubated for 30 min at 4 C in the dark. Finally, stained PBMCs were detected by FACSCalibur flow cytometer (Becton Dickinson, USA), and at least 50,000 events were recorded for each sample, and the data were analyzed using FlowJo software 10.0 (TreeStar, Ashland, U.S.A.). Fluorescence minus one control was utilized for the CD56 and CD16 antibodies. The lymphocyte population was first gated in forward scatter/side scatter plots, further CD3 ${\;}^{-}$ cells were selected in this population. Finally, CD3 ${\;}^{-}$ , CD16 ${\;}^{+}$ , and CD56 ${\;}^{+}$ cells were considered as NK cells.

### Enzyme-linked immunosorbent (ELISA) assay

The concentration of IFN-γ in the serum samples was quantified using an IFN-γ ELISA kit (Karmania Pars Gene, Iran), and the 25(OH) vitamin D concentration was measured using an ELISA kit (Euroimmun Co., Germany) according to the manufacturer's instructions.

### Ethical considerations

All participants in the study gave their informed consent, and the protocol was authorized by the Ahvaz Jundishapur University of Medical Sciences Ethics Committee, Ahvaz, Iran (Code: IR.AJUMS.MEDICINE.REC.1400.053).

### Statistical analysis

The normal or non-normal distribution of data were checked using the Kolmogorov-Smirnov test. The independent *t* test was used to analyze normal data, whereas the Mann-Whitney test was used for non-normal data. Statistical comparative tests were performed using the Mann-Whitney method, and Spearman's non-parametric method was used to check the correlation. The Chi-square test of Independence was used to evaluate if 2 categorical variables were associated. All analyses were performed at the p-value ≤ 0.05 error level and with the help of GraphPad Prism 9.3.0 software. The results are reported as Mean ± SEM (standard error of mean).

## 3. Results

### Clinical characteristics of the participants

59 women were enrolled in the study, including 29 women with endometriosis and 30 healthy women as the control group. No statistically significant difference was observed in the age and body mass index of the studied women (Table I). According to table I, more women in the case group compared with the control group experienced dysmenorrhea (68.96 vs 36.66). Also, symptoms such as dyspareunia (p < 0.001) and pelvic pain (p < 0.001) were significantly higher in case group than in the control group. Table II shows the mean percentage of NK cells and serum levels of IFN-γ and vitamin D in the 2 study groups.

### Evaluation of NK cells population 

**Figure 1:**
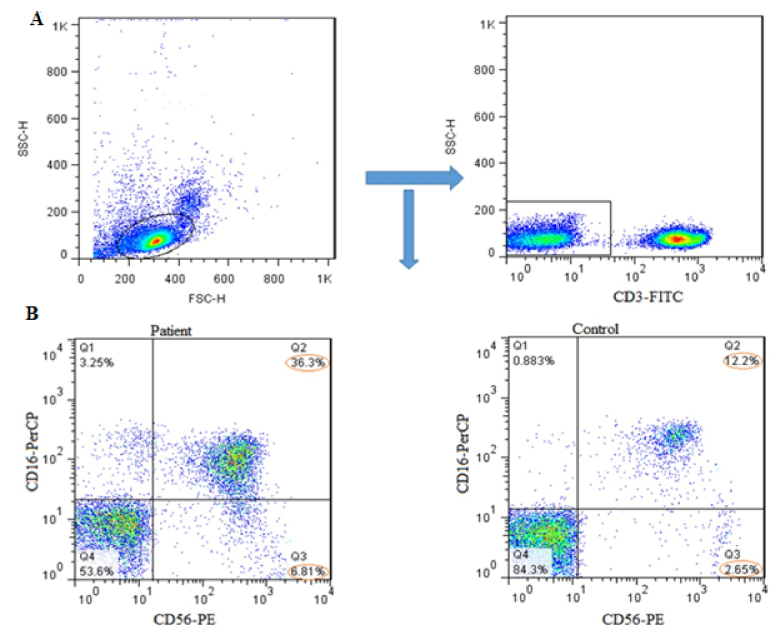
A) Shows the steps taken to isolate first) lymphocyte gate from the total cell population and second) CD3- cells from the rest of lymphocyte population using anti-CD3 antibody. B) Anti-CD56 and anti-CD16 antibodies were used to detect CD3-CD56+CD16+ (NK cells) population in case (patient) and control groups.

**Figure 2:**
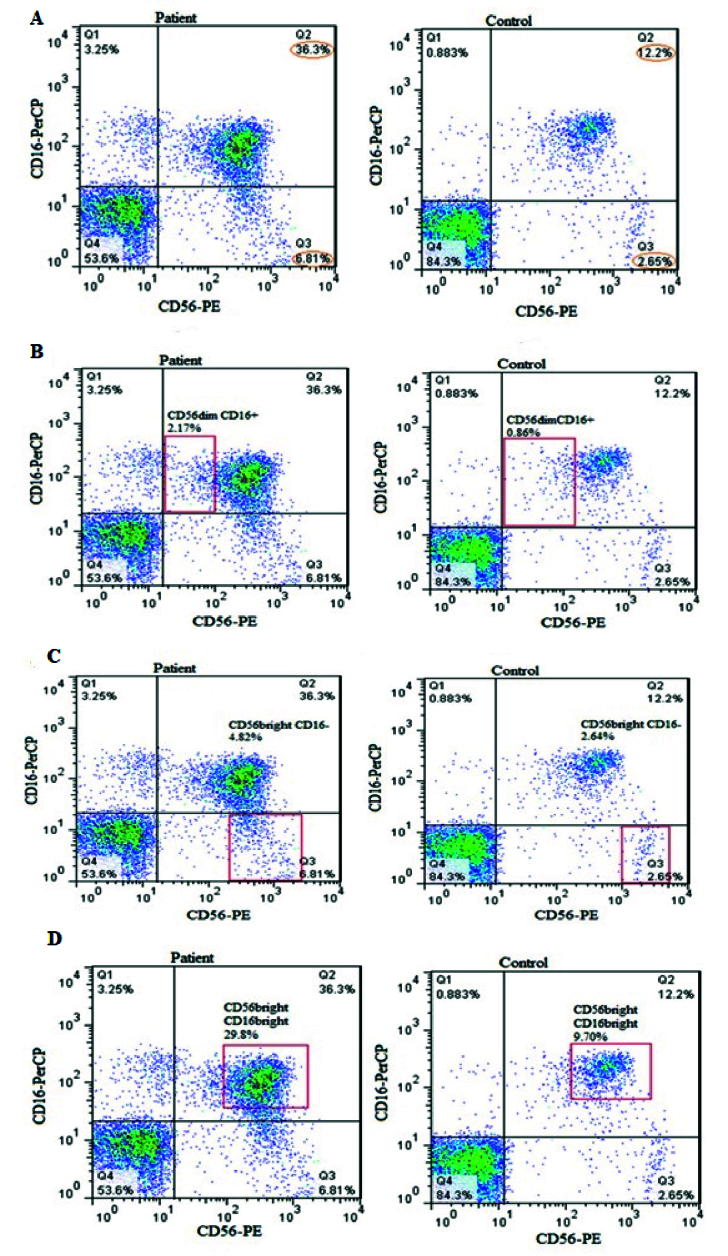
A) The percentage of NK cells in the PB of women with endometriosis and the healthy controls, which showed a significant increase in NK cells in case group compared to the control group (p = 0.03). B) The percentage of CD56 ${\;}^{\text{dim}}$ CD16 ${\;}^{+}$ NK cells subpopulation in women with endometriosis and healthy control group, which showed a significant increase in CD56 ${\;}^{\text{dim}}$ CD16 ${\;}^{+}$ NK cells subpopulation in case group compared to the control group (p = 0.007). C) The percentage of CD56 ${\;}^{\text{bright}}$ CD16 ${\;}^{-}$ NK cells subpopulation in women with endometriosis and the healthy control group. No statistical difference was observed between the case and control groups (p = 0.383). D) The percentage of CD56 ${\;}^{\text{bright}}$ CD16 ${\;}^{\text{bright}}$ NK cells subpopulation in women with endometriosis and healthy control group, which showed a significant increase in CD56 ${\;}^{\text{bright}}$ CD16 ${\;}^{\text{bright}}$ NK cells subpopulation in the case group compared to the control group (p = 0.043).

**Figure 3:**
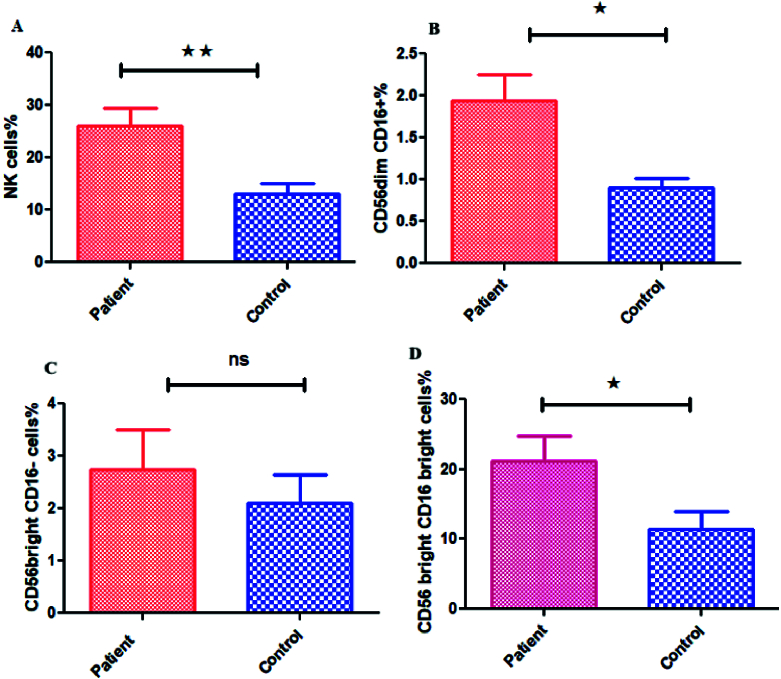
A)Comparison of mean percentage of NK cells in women with endometriosis and the healthy controls. B) Comparison of mean percentage of CD56 ${\;}^{\text{dim}}$ CD16 ${\;}^{+}$ NK cells subpopulation in women with endometriosis and healthy control group. C) Comparison of the mean percentage of CD56 ${\;}^{\text{bright}}$ CD16 ${\;}^{-}$ NK cells subpopulation in women with endometriosis and the healthy control group. D) Comparison of mean percentage of CD56 ${\;}^{\text{bright}}$ CD16 ${\;}^{\text{bright}}$ NK cells subpopulation in women with endometriosis and the healthy control group (*Significant at level of 0.05, **Significant at level of 0.01, ns: Not significant).

For determining the NK cells population, lymphocyte gates in the total cell population were isolated, and then in the CD3 ${\;}^{-}$ cells population we selected CD16 ${\;}^{+}$ and CD56 ${\;}^{+}$ cells as NK cells (Figure 1A, B). The mean percentage of the total NK cells population (in the CD3 ${\;}^{-}$ lymphocyte population) in the case group showed a significant increase compared to the controls (p = 0.03) (Figure 2A, 3A).

Also, a significant increase was observed in NK cells subpopulation in case group compared to the healthy control group, including CD56 ${\;}^{\text{dim}}$ CD16 ${\;}^{+}$ (p = 0.007) (Figure 2B, 3B) and CD56 ${\;}^{\text{bright}}$ CD16 ${\;}^{\text{bright}}$ NK cells subpopulation (in CD3 ${\;}^{-}$ lymphocyte population) (p = 0.043) (Figure 2D, 3D). However, no significant difference was observed in CD56 ${\;}^{\text{bright}}$ CD16 ${\;}^{-}$ subpopulation (p = 0.383) (Figure 2C, 3C). Also, no difference was observed in NK cells (p = 0.072) percentage in the endometriosis subjects according to variations related to the phase of the menstrual cycle.

### Comparison of serum IFN-γ and vitamin D 

**Figure 4:**
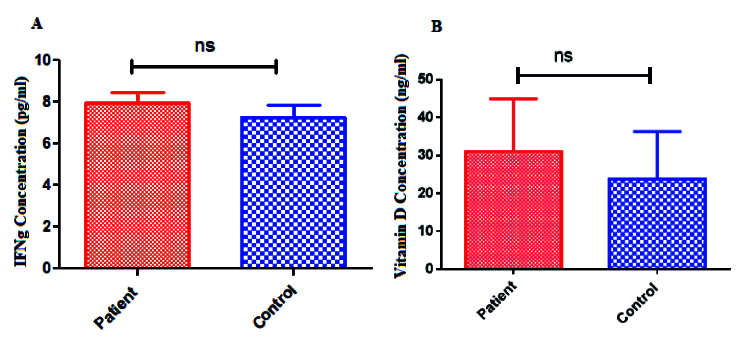
A)Serum levels of IFN-γ in women with endometriosis and the healthy controls. No statistical difference was observed between the cases and control groups (p = 0.41). B) Serum levels of vitamin D in women with endometriosis and the controls. No statistical difference was observed between the cases and control groups (p = 0.12) (ns: No significant).

The serum level of IFN-γ increased in women with endometriosis compared to the control group, but no statistically significant difference was seen (p = 0.41) (Figure 4A). Although the serum level of vitamin D in women with endometriosis was increased compared to the control group, it was not statistically significant (p = 0.12) (Figure 4B).

### Correlation between NK cells percentage and serum levels of IFN-γ in the endometriosis group

**Figure 5:**
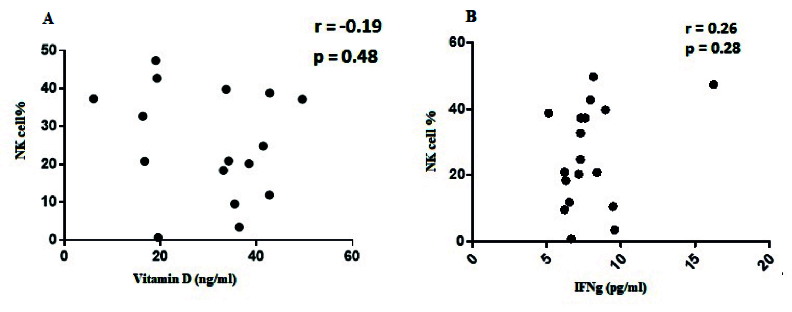
A) There is no correlation between NK cells and serum levels of IFN-γ in the study groups (r = 0.26, p = 0.28). B) No correlation between NK cells and serum levels of vitamin D in the study groups (r = -0.19, p = 0.48).

Spearman's correlation test was performed to investigate the association between NK cells and IFN-γ serum concentration. The result showed that no correlation was observed between the percentage of NK cells and the serum level of IFN-γ (r = 0.26, p = 0.28) (Figure 5A). Also, no correlation was observed between the percentage of NK cells subpopulation CD56 ${\;}^{\text{dim}}$ CD16 ${\;}^{+}$ (r = 0.201, p = 0.42) and CD56 ${\;}^{\text{bright}}$ CD16 ${\;}^{\text{bright}}$ (r = -0.43, p = 0.07), and the serum level of IFN-γ in the studied group. However, a significant positive correlation was observed between the percentage of NK cells subpopulation CD56 ${\;}^{\text{bright}}$ CD16 ${\;}^{-}$ , (r = 0.631, p = 0.007), and the serum levels of IFN-γ in the studied group.

### Correlation between NK cells percentage and serum vitamin D level in the endometriosis group

No correlation was observed between the percentage of NK cells and serum vitamin D level in the studied group (r = -0.19, p = 0.48) (Figure 5B). Also, no correlation was observed between the percentage of CD56 ${\;}^{\text{dim}}$ CD16 ${\;}^{+}$ (r = 0.306, p = 0.217), CD56 ${\;}^{\text{bright}}$ CD16 ${\;}^{-}$ (r = -0.016, p = 0.951), and CD56 ${\;}^{\text{bright}}$ CD16 ${\;}^{\text{bright}}$ (r = 0.265, p = 0.287) NK cells subpopulation and serum vitamin D level in the studied group.

**Table 1:** Demographic and clinical information of the participants


**Characteristics**		Case	**Control**	**P-value**
Age (yr)*		36.24 ± 5.62	35.90 ± 5.38	0.813
BMI (kg/m^2^)*		26.96 ± 4.73	25.76 ± 3.08	0.826
Working status**				
	**Employed**	7 (24.14)	11 (36.67)	
	**Unemployed**	22 (75.86)	19 (63.33)	0.399
Phase of cycle**				
	**Proliferative, days 1–14**	15 (51.72)	18 (60)	
	**Secretory, days 15–30**	14 (48.28)	12 (40)	0.604
Previous deliveries**				
	**None**	6 (20.69)	0 (0)	
	**1**	12 (41.38)	14 (46.67)	
	**≥ 2**	11 (37.93)	16 (53.33)	0.029
Dysmenorrhea**		20 (68.96)	11 (36.66)		0.019
Dyspareunia**		15 (51.72)	0 (0)		< 0.001
Pelvic pain**		16 (55.17)	0 (0)	< 0.001
*Data presented as Mean ± SD, Independent samples *t* test. **Data presented as n (%), Pearson's Chi-squared test. BMI: Body mass index				

**Table 2:** The mean percentage of NK cells and serum levels of IFN-γ and vitamin D in the 2 study groups


**Characteristics**	**Control**	Case	**P-value**
**Total NK cells%**	15.12 ± 2.85	25.94 ± 3.59	0.03
**CD56 ${\;}^{\text{dim}}$ CD16 ${\;}^{+}$ NK cell%**	0.9 ± 0.4	1.93 ± 1.29	0.007
**CD56 ${\;}^{\text{bright}}$ CD16 ${\;}^{-}$ NK cell%**	1.61 ± 0.96	2.12 ± 1.86	0.383
**CD56 ${\;}^{\text{bright}}$ CD16 ${\;}^{\text{bright}}$ **	11.37 ± 2.57	21.12 ± 3.67	0.043
**IFN-γ (pg/ml)**	7.277 ± 0.60	7.978 ± 0.46	0.41
**Vitamin D (ng/ml)**	23.71 ± 3.35	30.98 ± 3.10	0.12
Data presented as the Mean ± SEM, Independent samples *t* test, NK: Natural killer, CD: Cluster of differentiation, IFN-γ: Interferon-gamma			

## 4. Discussion

In this study, we observed that the total NK cells population in the case group was significantly increased compared to the control group. Also, a significant increase in CD56 ${\;}^{\text{dim}}$ CD16 ${\;}^{+}$ and CD56 ${\;}^{\text{bright}}$ CD16 ${\;}^{\text{bright}}$ NK cells subpopulation was detected in endometriosis individuals compared to the control group. Consistent with our findings, it was reported that the mean percentage of NK cells in patients with endometriosis is significantly higher than in healthy individuals (12). NK T cells, another important factor in innate immunity, showed a significantly lower percentage in endometriosis cases compared to control group in the study. Different studies have reported contradictory results regarding the frequency of NK cells in PB and peritoneal fluid, with decreases or lack of differences between women with endometriosis and controls (2). It should be noted that in the study of Schmitz et al., they did not observe any significant difference in NK cells subsets of PB or menstrual effluent between endometriosis individuals and control group (13). Also, in our study, no difference was observed in NK cells percentage in the endometriosis subjects according to variations related to the phase of the menstrual cycle, which is in accordance with the other similar study (14). Despite the increase of NK cells in the PB and endometrium of individuals with endometriosis compared to the healthy control group, a decrease in the activity of these cells has been reported, and a huge decrease in the activity of these cells occurs at the advanced stages of endometriosis (15, 16).

NK cell functions are controlled by a variety of killer-activating receptors and killer-inhibitory receptors (KIR). Positive and negative signal transmission in NK cells happen through these receptors, respectively. KIR molecules are a multigenic family of immunoglobulin-like families that realize major histocompatibility complex class I and suppress NK cell activation (17). The increased expression of KIR in peripheral NK cells has been indicated in endometriosis subjects (18). Upregulation of inhibitory receptors on the cell surface of NK cells has been indicated in endometriosis individuals compared to healthy subjects (5). Increased expression of inhibitory receptors in NK cells may be the reason for the decreased activity of these cells. These reports reflect that with less activity from NK cells, the immune system tries to increase the number of these cells to prevent endometriosis. Studies have demonstrated that Th17 cells and IL-17A have essential functions in the onset of endometriosis. These cells promote the survival of endometrial cells and make them resistant to NK cell cytotoxicity. This happens through the activation of ERK1/2 signaling (19).

It was shown that IFN-γ stimulates apoptosis in eutopic endometrial cells but not in ectopic endometrial cells and may be the cause of the uncontrolled growth of endometriotic lesions (20). In addition, it has been shown that IFN-γ increases the expression of intracellular adhesion molecule-1 and may be related to the defective function of NK cells in endometriosis (21). Ectopic endometrial cells probably evade the cytotoxic mechanisms of NK cells by inhibiting.

In the present study, the serum levels of this cytokine increased in women with endometriosis compared to the control group, but it was not statistically significant. Our results regarding the concentration of IFN-γ in PB samples were consistent with another study (22). The author from another study reported that despite observing a significant increase in the levels of IFN-γ in the peritoneal fluid of individuals with endometriosis compared to the control group, they did not observe any difference in the level of cytokine IFN-γ in the PB of the cases compared to the control group (23). However, conflicting reports have been published regarding the concentration of this cytokine between endometriosis and non-endometriosis cases (8, 24). Additionally, it was shown that the expression of NKp46 and its co-expressed activating receptors are altered in endometriosis, leading to a reduction in NK cell cytotoxicity. As a result, endometrial cells in the abdominal cavity may not be eliminated by NK cells, leading to the production of TNF-α and IFN-γ (25).

Vitamin D deficiency is a predisposing factor for various diseases in women, such as uterine fibroids, endometriosis, breast cancer, recurrent pregnancy loss, and in vitro fertilization failure (26, 27). Vitamin D regulates immune responses through its receptor, which is located on the surface of most immune cells, including lymphocytes. It can affect the production of IL-4, IL-5, IL-6, IL-10, IL-13, and IFN-γ cytokines. According to the studies, it has been determined that vitamin D plays a role in the development, maturation, differentiation, and cytotoxicity of NK cells (9).

Irrespective of the presence or absence of cytokines, vitamin D decreases NK cells cytotoxicity while increasing inhibitory receptor expression and decreasing activation receptors. It also prevents the lethal activity of cytotoxic T cells and NK cells by reducing the polarization of perforin on the surface of cells. Accordingly, it has been reported that NK cells' function is greatly influenced by vitamin D (28). We observed a rise in serum vitamin D levels in the PB of women with endometriosis compared to the control group, but this was not statistically significant. Consistent with our findings, other studies did not observe any difference in serum vitamin D levels between cases with endometriosis and healthy subjects. In another study, endometriosis women with treatment showed higher levels of 25(OH) vitamin D in their serum compared with those without (29). Moreover, there are conflicting reports regarding serum vitamin D concentrations between endometriosis and non-endometriosis cases (30). Differences in vitamin D measurement methods, receptor polymorphism, and binding protein may contribute to varying reports of vitamin D's role in endometriosis.

In this study, we focused on measuring the amount of NK cells and different subtypes of NK cells, as well as examining the correlation of these NK cell subsets with vitamin D and IFN-γ. However, the study has limitations. We only measured the amount of NK cells in PB, not the endometrial tissue. Additionally, we measured the level of IFN-γ in serum, not intracellularly. Finally, we did not investigate the vitamin D receptor at the level of immune cells.

## 5. Conclusion

In conclusion, we found that the percentage of total NK cells, CD56 ${\;}^{\text{dim}}$ CD16 ${\;}^{+}$ and CD56 ${\;}^{\text{bright}}$ CD16 ${\;}^{\text{bright}}$ NK cell subsets, were significantly increased in women with endometriosis. In addition, the level of serum vitamin D and IFN-γ increased in women with endometriosis compared to control group. However, no significant difference was observed. According to these findings, the study of the function and percentage of NK cell subsets can be useful in assessing and treating endometriosis disease. However, more comprehensive studies in the future are required to draw definitive conclusions about these observations.

##  Data availability

Data associated with the paper is available upon reasonable request from the corresponding author.

##  Author contributions

S.NCh: Conceptualization and methodology and writing original draft. M.Gh: Conceptualization and supervision, and writing-review and editing. N.E: Flow cytometry analysis. A.A: Software and validation. M.S: Formal analysis. M.H: Taking endometrial biopsies. All authors read and approved the final manuscript.

##  Conflict of Interest

The authors declare that there is no conflict of interest.
